# Pediatric recurrent respiratory tract infections: when and how to explore the immune system? (About 53 cases)

**DOI:** 10.11604/pamj.2016.24.53.3481

**Published:** 2016-05-12

**Authors:** Mohammed El-Azami-El-Idrissi, Mounia Lakhdar-Idrissi, Sanae Chaouki, Samir Atmani, Abdelhak Bouharrou, Moustapha Hida

**Affiliations:** 1 Laboratory of Immunology, Faculty of Medicine and Pharmacy at Sidi Mohamed Ben Abdellah University of Fez, Hassan II University Hospital of Fez, Morocco; 2 Department of Pediatrics, Faculty of Medicine and Pharmacy, Sidi Mohamed Ben Abdellah University of Fez, Hassan II University Hospital of Fez, Morocco

**Keywords:** Recurrent respiratory infections, Pediatrics, Immune deficiency

## Abstract

Recurrent respiratory tract infections are one of the most frequent reasons for pediatric visits and hospitalization. Causes of this pathology are multiple ranging from congenital to acquired and local to general. Immune deficiencies are considered as underlying conditions predisposing to this pathology. Our work is about to determine when and how to explore the immune system when facing recurrent respiratory infections. This was based on the records of 53 children hospitalized at the pediatrics unit of Hassan II University Hospital, Fez Morocco. Thirty boys and 23 girls with age ranging from 5 months to 12 years with an average age of 2 years were involved in this study. Bronchial foreign body was the main etiology in children of 3 to 6 year old. Gastro-esophageal reflux, which in some cases is a consequence of chronic cough, as well as asthma were most frequent in infants (17 and 15% respectively). Immune deficiency was described in 7.5% of patients and the only death we deplored in our series belongs to this group. Recurrent respiratory tract infections have multiple causes. In our series they are dominated by foreign body inhalation and gastroesophageal reflux, which in some cases is a consequence of a chronic cough. Immune deficiency is not frequent but could influence the prognosis. Therefore immune explorations should be well codified.

## Introduction

Recurrent respiratory infections have multiple etiologies. They are frequent during childhood and seem inevitable during the first months of life because of the immunity learning. A good knowledge of these aspects is necessary in order to differentiate between a physiological (immunity learning) and a pathological (immune deficiency) situation [1].

Recurrence of respiratory infections during the first years of life has an impact on the broncho-alveolar as well as the vascular development of the lungs. This could lead to average and long term after-effects. Therefore, an early treatment, depending on the etiology, should be initiated. According to World Health Organization (WHO) data, a kid could present, annually, during its five years of life, 4 to 8 episodes of respiratory infections, affecting mainly lower respiratory system. Respiratory infections are considered as recurrent from three episodes of acute infections during a six month period [2].

## Methods

This work is about a retrospective, descriptive and analytical study, including 53 cases of recurrent low respiratory infections brought together at the department of pediatrics of the University Hospital Hassan II of Fez, during a 3 years period (January 2009 - December 2011). All cases of recurrent high respiratory tract infections as well as non hospitalized cases were not included in this study. The cases of pulmonary tuberculosis were also eliminated because often associated with a persistent chronic cough.

We carried out a descriptive study of ours series and an analytical study of the immune deficiency cases concerning the notion of consanguinity, the family history, the age, the sex, the clinical presentation and the evolution.

## Results

The age of the children is between 5 months and 12 years. The average age is of 2 years. There were 30 boys and 23 girls and more than half our patients (64.4%) are infants. The breast-feeding average duration was of 6 months (>2 children were not breast-fed). All patients were well vaccinated according to the national program of immunization. Clinical history in our series were marked by the chronic vomiting (13 patients), atopy ( 14 patients), passive smoking (9 patients). A penetration syndrome due to tracheobronchial foreign body aspiration was noted in 12 patients. The physical signs were mainly, cough (90%), fever (76%) and dyspnea (50%). 25% of our patients presented an altered general condition with one case of hemodynamic instability. The failure to thrive was found in 12 patients. The pleuro-pulmonary examination revealed 4 cases of chest deformities, low saturation in 10 patients, and acute respiratory distress in 18 children with a finger clubbing in 3 of them. The ear, nose and throat (ENT) examination found chronic rhinitis in 5 cases, recurrent purulent otitis media in one child and a tonsillitis in 2 cases.

**Figure 1:**
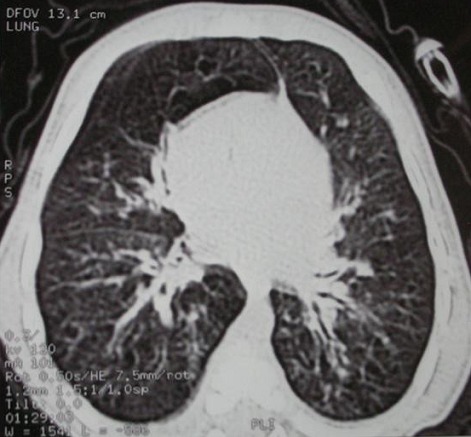
Chest CT scan showing bronchiectasis in a patient suffering from cystic fibrosis

Chest X-ray, performed for all patients, showed localized lesions in 18 cases, bilateral lesions in 31 cases (alveolar syndrome in 12 cases, bronchial syndrome in 17 cases, and interstitial syndrome in 2 cases) and was normal in 4 cases. Chest CT scan revealed a foreign body in 3 cases, a diaphragmatic hernia in one case and a bronchiectasis in 6 cases (Figure 1). Upper gastrointestinal series radiography in 7 patients showed gastro-esophageal reflux in 3 patients with a hiatus hernia in two of them. Esophageal pH monitoring performed in 6 patients with no signs of gastroesophageal reflux disease (GERD) showed reflux in 4 cases (other than those revealed by upper gastrointestinal series). Reflux was important in two cases, moderate in one case and minimal in another. The relationship between episodes of cough and reflux wasn't established.

**Figure 2:**
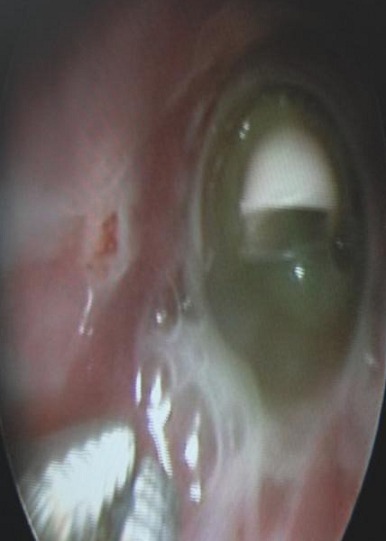
Whistle of a toy at the right main bronchus

Thirteen patients underwent bronchoscopy as part of a foreign body exploration (9 for diagnostic purposes), and these patients benefited from an endoscopic extraction (Figure 2). The upper gastrointestinal endoscopy performed in three patients presenting chronic vomiting associated with recurrent bronchopneumonia revealed one case of stage 1 and one case of stage 2 esophagitis.

Biologic abnormalities were also found: 1) 25 patients have a microcytic hypochromic anemia which was iron-deficiency in 18 of them; 2) leukocytosis was shown in 21 patients with neutrophil predominance in 16 of them; 3) eosinophilia was found in 3 patients; 4) C-reactive protein (CRP) had a value between 5 and 360 mg/l with an average of 74; and 5) the detection of *Mycobacterium tuberculosis* and the tuberculin skin testing performed for 13 patients were negative in all cases. HIV serology was negative in all patients for whom it was performed. Constitutional immune deficiency was searched for in 11 patients and was found only in 4 cases (Table 1). T lymphocytes subsets were determined in one patient and were normal. Total serum IgE was measured in 4 patients and was also normal. The specific IgE RAST test was performed in one patient and was negative. No allergy skin test was performed in our series and the sweat test was positive in 3 of the 7 patients for whom it was performed.

**Table 1:** Results of the immune system evaluation in our patients

Immune test	Number of patients tested	Number of positive cases	Anomaly /Number of cases	
HIV serology	8	0	**Anomaly**	**Number of cases**
Immunoglobulin dosage	11	4	IgA deficiency	3
			IgA and IgG deficiency	1
Lymphocytes subsets	1	0		
Total serum IgE dosage	1	0		
Specific IgE determination by RAST	1	0		
Allergy skin test	0	0		

After all these investigations, a cause of recurrent respiratory infections was found for 43 patients (81.1%). Causes according to the age are summarized in (Table 2) and the relationship between age and etiology was significant (p = 0.014).

**Table 2:** Recurrent respiratory infections causes in our patients depending on the age

Age Cause	Infant		Child of 3 to 6 years		Child of 7 to 12 years	
	Number of cases	%	Number of cases	%	Number of cases	%
Bronchial foreign body	6	17.1	7	46	0	0
Gastroesophageal reflux	7	20	3	20	0	0
Asthma	7	20	0	0	2	22
Immune deficiency	3	8.5	1	6.6	0	0
Swallowing disorder	3	8.5	0	0	0	0
Cystic fibrosis	3	8.5	0	0	0	0
Rickets	1	2.8	0	0	0	0

Immune deficiency represents 9.3% of the causes and was found in 7.5% of all cases. It was more common in infants less than 3 years of age and was predominant in boys (3/1). Consanguinity was noted in 2 cases. One patient had a case of death in his siblings by repetitive infections. The immune deficiency was accompanied by respiratory distress in one case and a failure to thrive in half of the cases (p = 0.65). The only death in this series of 53 patients was an infant of six months carrying a combined immunedeficiency of IgA and IgG.

## Discussion

Respiratory infections may recur in infants and children mainly for many reasons. The involvement of immune deficiencies is certainly weak compared to other etiologies and it is exceptional that an immune deficiency is revealed only by recurrent respiratory infections, without association with infections of other locations [2].

It is normal to have several respiratory infections in early life because of immune immaturity. The highest incidence is between six and 18 months. These infections (like other early infections) help to train the immune system. The immune immaturity leads to ENT carriage of bacteria like *Streptococcus pneumonia* [3]. Although transient in nature, the concept of delayed maturation of the immune system is important because these children could benefit clinically from extra stimulation like vaccination [4].

Other children are unfortunately unable to respond to even optimal vaccine stimulation. In practice, it is convenient to distinguish between ordinary recurrent respiratory infections due to the immune learning and pathological respiratory infections which appear, often, to be linked with a particular cause that should be looked for (Table 3).

**Table 3:** The criteria for the diagnosis of infections related to the phenomenon of immune learning

Nature of infection	Ordinary (rhinitis, rhino-pharyngitis, rhino-bronchitis
Frequency	Should not exceed 5 to 6 infections per a year (10 infections in some cases) with a trend of decrease with age
Season	Infections resolve completly or patialy during summer
signs «negative »	Between infectiuos episodes : Satisfactory general condition, No failure to thrive No other symptoms: chest wheezing (asthma), polypnea (asthma, cystic fibrosis), vomitting (GERD), diarrhea or alternating diarrhea-constipation (cystic fibrosis), mucosal or skin infections, extensive and superinfected eczema (immune deficiency).
Chest X-ray	Generally normal

Apart from the concept of immune immaturity, other immune causes are also known. The lack of breast milk feeding during the first six months, an important source of secretory IgA, lysozyme and lactotransferrin, increases the risk of infections including respiratory infections as well as the risk of allergy [5–7]. Malnutrition causes immunological disturbances that could lead to susceptibility to ENT infections as well as to lower respiratory tract, intestinal and skin infections.

The syndrome of passive inhalation of tobacco smoke (PITS) is manifested by a chronic cough (75%), recurrent bronchitis (33%) and ENT infections (13%) [8]. Immune explorations show a total serum IgE increase during PITS while skin allergy tests and specific IgE assays remain negative. This immune anomaly disappears few months after cessation of exposure to tobacco smoke. The mechanisms of the deleterious effects of PITS are numerous and entangled such as mucosal irritation, increased mucosal permeability, decreased migration of macrophages and neutrophils, increased total serum IgE. Children living in community have high infection risk especially viral infections (nasopharyngitis, laryngitis, bronchitis, bronchiolitis). This is also the case for bacterial infections (otitis and conjunctivitis) as evidenced by the higher frequency of pharyngeal carriage of the three main bacteria (*H. influenza, M. catarrhalis, S pneumonia*) in children kept in nursery. In addition, the infectious agents carried by kindergarten children have increased antibiotic resistance especially *S. pneumonia* [9]. Air pollution increases bronchial hyper-responsiveness and promote respiratory infections. The relationship between the importance of air pollution and respiratory allergic disease is well established [10].

### Why to explore child recurrent low respiratory infections?

Several studies have demonstrated that a remarkably high proportion of children with recurrent infections have abnormalities in the humoral immune system. Several decades ago, Schur et al. described selective immunoglobulin G (IgG) subclasses deficiencies in patients with pyogenic pulmonary infections [11].

Many long-term consequences may occur due to recurrent infections of lower respiratory tract and lead to significant chronic problems such as the appearance of bronchiectasis or antibiotic resistance. According to some authors, it has been shown that a thorough investigation of children with recurrent infections of the lower respiratory tract can identify, in at least 90% of cases, an underlying cause as it was the case in our series [12, 13].

This allows, at least in some situations, to provide prevention and treatment to these children. Clinicians should be encouraged to persevere in the etiological assessment. It has been shown through our series that favorable evolution was higher when the etiology was known and etiological treatment administered (69% versus 40%). A bronchial dilation was noted in 6 cases (11.3%), one of whom was diagnosed with cystic fibrosis.

### When to explore the immune system?

In our series, initial assessment was made in order to exclude etiologies easy to diagnose by physical or radiological examination. The immune exploration, difficult and expensive in our context, was not systematic but guided by a set of anamnestic and clinical criteria such as the notion of consanguinity, the presence of similar recurrent disease in a parent or sibling of the child as well as the association with other infections or other signs such as eczema, chronic diarrhea, recurrent oropharyngeal candidiasis or autoimmune manifestations. HIV serology was achieved accordingly in case of parental consent.

### How to explore the immune system?

Anamnesis of infections limited to respiratory tract makes the diagnosis of cellular immunity or phagocytosis deficiency unlikely. Humoral immunodeficiencies are most frequently responsible for recurrent bronchopneumopathies and start in a delayed manner in the first year of life (uncommon before 7 to 9 months of life) thanks to the persistence of maternal IgG. IgA deficiency is the most common humoral immunodeficiency as it was noted in our series. It has a prevalence of about 1/600 [14, 15].

There is an increased susceptibility to recurrent infections with encapsulated and pyogenic extracellular bacteria. It is rarely symptomatic by itself and there is no predisposition to fungal or viral infections. An association with IgG subclasses deficiency could reflect a deeper immune deficiency. The dosage of IgG subclasses is interpreted after the age of 18 months. It covers the IgG 1, 2 or 3 since the absence of IgG4 has no known pathological significance [16]. When accompanied by an IgA deficiency, finding a complex underlying primary immunodeficiency, such as ataxia telangiectasia, should be considered and a serum alpha-fetoprotein measurement as well as a lymphocyte phenotyping and oriented karyotyping can be achieved [14].

Antibody responses to previous antigenic exposure can also be measured. In fact, children who have been vaccinated in infancy against germs such as pneumococcus or *Haemophilus influenza* type B should have protective antibodies at the time of evaluation. Selective deficiency in the production of antibodies directed against polysaccharide antigens exposes to encapsulated bacteria infections. Recent studies have shown that specific antibody deficiency is common in young children with recurrent respiratory infections; but it is often transient and resolves by itself within few years without specific treatment [17, 18].

Indeed, the memory B cell compartment is already established at the age of 2 years in healthy children and those with recurrent respiratory infections and the decline of antibody may predispose children to recurrent infections in the absence of revaccination [19].

The X-linked agammaglobulinemia (Bruton′s disease) is often noisy with bacterial pneumonia willingly accompanied by bronchiectasis. The IgG, IgA and IgM levels are well below the normal values or close to zero. Circulating CD19 + CD20 + B cells are not detected.

The hyper-IgM syndrome is either an inherited X-linked PID due to mutations in the gene encoding CD40 ligand or autosomal recessive PID due to mutations in CD40, AID or UNG genes. It is defined by the absence of isotype switching and is characterized by the absence of IgG, IgA, IgE synthesis and normal or elevated IgM level. Opportunistic infections can reveal this PID and autoimmunity may be associated [20].

Common variable immunodeficiency is overwhelmingly accompanied in older children and young adults with symptoms similar to those of Bruton′s disease. There is a lack of cooperation between T and B lymphocytes characterized by an absence of differentiation of B lymphocytes towards plasma cell. There is an increased risk of cancers (lymphoma) and autoimmune diseases. The presence of an eczema can reveal it.

A deficiency of cellular immunity should always be considered in any opportunistic infections such as Pneumocystis carinii pneumonia or pulmonary aspergillosis or a severe viral infection. Recurrent respiratory infections due to common germs are generally not related to a T cell dysfunction and the exploration of T lymphocytes is required in a second time, apart from a possible screening for HIV.

A simple blood count can guide the diagnosis if there is a global lymphopenia. Anemia, thrombocytopenia or neutropenia may be associated with a cellular immune deficiency often in the context of autoimmunity. Neutropenia, by itself, can explain recurrent respiratory infections only if it is profound (<500/mm3) and prolonged. It is convenient to look for a cellular immune deficiency by phenotyping T lymphocytes, B lymphocytes and Natural killer cells in the whole blood by flow cytometry [14, 21]

Chronic granulomatous disease (CGD) is an inherited immune disorder characterized by a failure to activate the respiratory burst due to a defect in superoxide-generating NADPH oxidase of phagocytes. This leads to severe recurrent infections and unexplained prolonged inflammatory reactions that may produce granulomatous lesions mainly in the lung. The pulmonologists (pediatrics or adult) need to be able to recognize different lung manifestations of CGD for a best diagnosis [22].

## Conclusion

Immune deficiency is not a frequent etiology for recurrent respiratory infections and the exploration of the immune system should be well codified since the prognosis depends on it. The main challenge of monitoring recurrent respiratory infections is to distinguish between patients with normal immune system development and those expressing immune deficiencies. Therefore, to make the management of this pathology easier, without multiplying biological tests and treatments, diagnosis should be based on both clinical history, physical exam and some selected biological tests.

### What is known about this topic

- The study of etiologic profile of children's recurrent respiratory infections has already been subject of several studies.
- In many recurrent respiratory infection situations, no definitive cause is found.
- Immune deficiencies are considered as underlying conditions predisposing to this pathology.

### What this study adds

- Immune deficiency is rarely the cause of recurrent respiratory infections mainly upper respiratory infections.
- Medical practices must be built on anamnestic and clinical data rather than empirical approach.
- To avoid abusive prescription of immune explorations, the benefit/cost of laboratory test balance should be considered.
